# Perceived barriers, facilitators and usefulness of a psychoeducational intervention for individuals with chronic musculoskeletal pain and depression in primary care

**DOI:** 10.3389/fpsyg.2023.1099419

**Published:** 2023-04-25

**Authors:** Catarina Tomé-Pires, Enric Aragonès, Concepción Rambla, Germán López-Cortacans, Elisabet Sánchez-Rodríguez, Antonia Caballero, Jordi Miró

**Affiliations:** ^1^Department of Psychology, Psychology Research Centre, Autonomous University of Lisbon, Lisbon, Portugal; ^2^Institut Universitari d’Investigació en Atenció Primària Jordi Gol (IDIAP Jordi Gol), Barcelona, Spain; ^3^Atenció Primària Camp de Tarragona, Institut Català de la Salut, Tarragona, Spain; ^4^Unit for the Study and Treatment of Pain—ALGOS, Department of Psychology, Research Center for Behavior Assessment, Universitat Rovira i Virgili (URV), Tarragona, Spain; ^5^Institut d’Investigació Sanitària Pere Virgili, Universitat Rovira i Virgili (URV), Tarragona, Spain; ^6^Chair in Pediatric Pain Universitat Rovira i Virgili (URV)—Fundación Grünenthal, Catalonia, Spain

**Keywords:** chronic musculoskeletal pain, pain psychoeducation, depression, primary care, self-management, qualitative study

## Abstract

**Background and aims:**

Self-management interventions have the potential to improve patient’ pain condition as they involve tasks aimed at managing symptoms and reducing interference with activities, mood and relationships due to pain. However, research on factors that facilitate or hinder pain self-management has overlooked patients with both chronic musculoskeletal pain and depression in primary care settings, also leaving unattended patient views on the usefulness of such programs. Thus, the main aim of this study was to gather meaningful information to help promoting adequate self-management. Specifically, it attempts to identify patients’ perceptions of barriers and facilitators of group-based psychoeducational intervention and to explore its perceived usefulness in promoting self-management.

**Method:**

This qualitative study explored perceived barriers and facilitators of a psychoeducational intervention for the management of chronic musculoskeletal pain and depression previously tested in a Randomized Control Trial. We conducted focus groups and individual interviews with fifteen adult patients with both chronic musculoskeletal pain and depression recruited from primary care centres in Tarragona province (Catalonia, Spain). A content thematic analysis was carried out to examine the data. This study followed the Consolidated Criteria for Reporting Qualitative Research (COREQ) guidelines.

**Results:**

Findings revealed that perceived barriers included lack of motivation, time constraints, pain, depression, ineffectiveness of pain-relief strategies and activity avoidance. Facilitators were having a supportive family/friends, the positive effects of self-management, high motivation, being a proactive patient. Peer support and identification, the positive effect of sessions, and free expression were highlighted as key elements of the psychoeducational intervention.

**Conclusion:**

The psychoeducational intervention was perceived as useful in promoting self-management practices. Barriers and facilitators in using self-management strategies were related, mainly, to internal personal characteristics of the patients being similar among different cultural backgrounds and distinct chronic conditions.

**Implications:**

These findings can help to guide clinicians in the development and implementation of more effective pain self-management interventions for patients with chronic pain and depression by attending to their needs and preferences.

## Introduction

Chronic pain is a major health problem that can have deleterious effects on individuals’ physical, social and psychological functioning (Breivik et al., 2013; Burke et al., 2015; Dueñas et al., 2016). It is estimated that between 30 and 50% of people with chronic pain have depression (Bair et al., 2003; Dueñas et al., 2015; Zartaloudi et al., 2020). Both chronic pain and depression are common and relevant conditions in primary care patients (Fernández et al., 2010; Serrano-Blanco et al., 2010; Barrett and Chang, 2016; Dueñas et al., 2016).

Effective pain self-management has been established as a key therapeutic goal for individuals with chronic pain (Lukewich et al., 2015; Gordon et al., 2017; Devan et al., 2018; Fu et al., 2018; López-López et al., 2020). Multidisciplinary pain programmes encourage patients to learn and use self-management skills to cope with pain and its effects on function (Loeser and Turk, 2001; Ehde et al., 2014; Takahashi et al., 2018, 2019; Vanhaudenhuyse et al., 2018; Vartiainen et al., 2019; Nees et al., 2020). Results from clinical-based studies (e.g., arthritis, back pain, neck pain, fibromyalgia) repeatedly show that self-management leads to better clinical outcomes (e.g., reduce pain intensity and psychological distress) (Newman et al., 2004; Barlow et al., 2009; Devan et al., 2018).

Previous studies have shown that common barriers to the use of pain self-management strategies are related to individuals’ internal personal characteristics, as for example, fear of movement due to pain (Austrian et al., 2005; Bair et al., 2009; Lukewich et al., 2015; Mann et al., 2017), low self-efficacy (Gordon et al., 2017), sustained motivation (Matthias et al., 2016; Gordon et al., 2017; Devan et al., 2018), over-reliance on medication (Bair et al., 2009; Gordon et al., 2017), pain and depression interference (Austrian et al., 2005; Jerant et al., 2005; Lukewich et al., 2015; Craner et al., 2016; Gordon et al., 2017; Mann et al., 2017; Devan et al., 2018) and also external, as poor patient-physician communication (Jerant et al., 2005; Lukewich et al., 2015; Gordon et al., 2017; Mann et al., 2017), time constraints (Austrian et al., 2005; Bair et al., 2009; Fu et al., 2018), lack of close support (Jerant et al., 2005; Bair et al., 2009; Gordon et al., 2017; Devan et al., 2018), and limited treatment options (Austrian et al., 2005; Jerant et al., 2005; Park et al., 2013). In contrast, facilitators to chronic pain self-management have been briefly addressed in the literature, reporting internal personal characteristics of patients as, for example, self-discovery (Devan et al., 2018), beliefs about treatment benefits (Park et al., 2013), high self-esteem (Craner et al., 2016), and external characteristics as supportive family or friends (Park et al., 2013; Lukewich et al., 2015; Craner et al., 2016; Matthias et al., 2016; Mann et al., 2017; Devan et al., 2018), having economic resources (Park et al., 2013), and access to healthcare services (Lukewich et al., 2015; Mann et al., 2017).

Although chronic pain is a common health problem encountered in primary care (Fernández et al., 2010; Serrano-Blanco et al., 2010; Gordon et al., 2017), programmes supporting self-management are often difficult to implement in such context (Warsi et al., 2003; Turk et al., 2008). Therefore, supporting self-management of chronic pain in primary care is challenging as optimal self-management may be difficult to achieve (Newman et al., 2004; Lukewich et al., 2015; Gordon et al., 2017). In fact, we recently noticed several challenges in the implementation and testing of a pain self-management multicomponent programme (RCT) conducted in primary care centres. Despite its clinical improvements (e.g., lower depression levels) we observed a low adherence to the intervention. Furthermore, a deep analysis is required to understand what facilitates and hampers self-management interventions in this specific setting calling into attention the need to consider a patient-centered approach (Jayadevappa and Chhatre, 2011; Lin et al., 2020).

A closer look to the literature on barriers and facilitators to pain self-management reveals several gaps and shortcomings. Therefore, this problem is still insufficiently explored. First, most studies have relied on samples of adults with various chronic pain conditions (e.g., Jerant et al., 2005; Park et al., 2013; Devan et al., 2018) making it difficult to distinguish whether recommendations are valid for specific chronic pain conditions. Second, only a few studies specifically investigated patients with chronic musculoskeletal pain attending primary care settings, including those with comorbid depression (Bair et al., 2009; Damush et al., 2016). Third, research in this field remains limited to Anglo-Saxon countries evidencing the need to explore more cultural backgrounds. Forth, studies reporting data on patients’ perceived usefulness of self-management programmes are scarce (Cuperus et al., 2013; Nees et al., 2020) and need further examination. Indeed, pain-related improvements have been found to be influenced by the patients’ perceived treatment helpfulness (Nees et al., 2020).

Based on these previous considerations and filling the research gap in barriers and facilitators to pain self-management, the present study has a 2-fold purpose: (1) to explore what worked (i.e., facilitators) and did not work (i.e., barriers) in the self-management intervention delivered to a sample of Spanish adults with chronic musculoskeletal pain and depression in primary care (Aragonès et al., 2016), and (2) to examine whether the intervention was found useful in promoting self-management practices. Contributions made from this study should be of wide interest and guide pain care practices of healthcare professionals dealing with chronic pain and depression in primary care settings.

## Materials and methods

### Design

This qualitative study is a further analyses after a previous RCT (Aragonès et al., 2016). In this study we used a qualitative descriptive approach and carried out qualitative interviews (i.e., focus groups and individual telephonic interviews), to gather and explore in more depth perceptions of barriers/facilitators and the usefulness of the psychoeducational intervention. As part of the RCT (Aragonès et al., 2019) this study had a clinical trial registration completed on ClinicalTrials.gov (NCT02605278).

### Setting

Data was collected in eight urban primary care centres located in Salou, Reus, and Tarragona (Catalonia, Spain). The total population of Catalonia is 7,747,709 (Institut d’Estadística de Catalunya, 2023). Catalonia, like other autonomous communities in Spain, has a well-developed public primary care system with universal coverage. This healthcare system operates following the Beveridge model, similar to countries such as United Kingdom, Italy, and Portugal (Salvador-Carulla et al., 2010). It functions via catchment areas and primary care centres staffed by a variety of healthcare professionals (e.g., family physicians/general practitioner, pediatricians, nurses). To ensure continuity and a long-term relationship with patients, healthcare professionals are assigned a stable list of individuals under their care. This primary care system is also linked to hospital outpatient clinics and community mental health centres, which provide specialized outpatient care through referrals from primary care (Borkan et al., 2010; Salvador-Carulla et al., 2010). Further, the psychologist (the first author of the study—CTP—with interest and experience in the pain field) who developed and implemented the psychoeducational programme was based in primary care centres. During the study period, research team members were mainly based in primary care centres and were Assistant Lecturers in Psychology (CTP and ESR), Full Professor (JM), GP physicians (EA,AC and CR) and a Registered Nurse (GLC).

### Intervention

The group-based psychoeducational intervention was based on a cognitive-behavioral orientation organized into nine-weekly sessions of 2 h led by a female psychologist (CTP). Details about the intervention are described in more detail elsewhere (Aragonès et al., 2016, 2019). This psychoeducational intervention was a key component of a multicomponent programme previously tested for the management of chronic musculoskeletal pain and depression (Aragonès et al., 2016). The programme also included: (1) optimized management of depression following a computerized clinical guide for depression and (2) care management activities (periodic clinical monitoring and support of patients). Psychoeducational group sessions took place in the participants’ reference primary care centres and included a presentation of the content to encourage interaction between participants, and the application of learned skills through practice outside the sessions. To engage participants in their own self-management process, we encouraged patients to take a proactive role. We asked them to put into practice some of the learnt exercises, (e.g., to practice breathing or distraction exercises) outside sessions (at home/work). “Homework” was assigned after each session, which was then reviewed at the beginning of each of the following sessions to check possible challenges they faced when performing it. The content of the psychoeducational sessions covered several areas considered relevant in the context of chronic pain management (see Table 1). To facilitate the sessions, a teaching manual was made available, along with other support materials (slide-based presentations, brochures, and forms) to all participants attending the sessions.

**TABLE 1:** Psychoeducational intervention program features.

Session number/Topic	Content/Aims
1. Pain	Understand pain as a multidimensional experience
	Encourage an active role in the management of pain
2. Managing attention and emotions	Understand the importance and effects of both attention and emotions in the experience of pain
	Promote the sensation of self-control upon pain through recognizing and managing emotions and attention by practising
3. Basic relaxation techniques	Understanding the vicious circle between pain and tension
	Understand the benefits of relaxation in pain; Learn to break the vicious circle between pain-tension-pain through learning and practising of basic relaxation techniques
4. Cognitive restructuring (CR) strategies (p.1)	Understanding the importance of thoughts in emotions and behaviors
	Learn to distinguish irrational negative thoughts from rational thoughts; Observe, identify, and register thoughts
5. CR strategies (p.2); reinforcement of activities	Identify and learn strategies to substitute negative automatic thoughts for adaptive thoughts; Identify and programming pleasurable activities to reinforce psychosocial wellbeing
6. Problem solving	Understand and learn how to apply the problem-solving technique in daily problems related to pain
7. Life goals	Identify life goals and plan how to accomplish them
8. Physical activity, postures, and sleep	Understand the importance of physical activity in pain (circle of inactivity – pain; pain-inactivity), its benefits and its planning
	Promotion of healthy postures and healthy sleepy habits
9. Maintenance and relapse plan	Look over the topics discussed during sessions
	Discuss accomplished achievements and learned useful strategies
	Establishment of a relapse plan to prepare the patient to possible relapses

### Data collection

In this study, we employed interviews as data the collection method (i.e., focus groups and individual interviews) to gain an in-depth understanding of the facilitators, barriers, and usefulness of the psychoeducational intervention as perceived by the participants. Interviews were carried out in a clinical setting, i.e., primary care centres.

### Participants and recruitment

Potential participants were recruited from the intervention arm of the RCT testing the clinical effectiveness of a multicomponent intervention in the management of chronic musculoskeletal pain and depression (Aragonès et al., 2016). The intervention arm of the RCT included 167 participants from whom 103 agreed to participate in the psychoeducational sessions. From those 103 participants, 51 attended at least half of the total number of sessions (i.e., 5 out of 9). Participants of this present study (*N* = 15) were adult patients, with chronic musculoskeletal pain of at least moderate severity [≥5 points in the severity subscale of the Brief Pain Inventory; BPI; (Cleeland and Ryan, 1994; de Andrés Ares et al., 2014) and diagnosed with major depression according to the DSM-5 (American Psychiatric Association, 2013), attending primary care centres in the province of Tarragona (Catalonia, Spain). The BPI is a questionnaire which measures pain intensity with four 0 to 10 numerical rating scales (0 = “*no pain*” to 10 = “*pain as bad as you can imagine*”). Respondents are asked to rate their current, worst, least, and average pain in the last week. An overall score of pain severity is obtained by averaging the four BPI pain intensity items].

Ten participants were recruited among those who fully participated in the original RCT and were split into two face-to-face focus groups (five in each group). The other five participants were recruited among patients who had been invited to the RCT but demonstrated little or no adherence to the psychoeducational programme (i.e., these were individuals who attended 0–2 session) and were interviewed individually by telephone. All participants were made aware of the researchers’ motivations for conducting the study.

### Focus groups

Participants (*N* = 10) in the focus groups were selected from the intervention group of the original study (Aragonès et al., 2016). A purposive sampling was used to have access to a particular subset of people with specific characteristics. Their selection was intended to be representative in terms of sex, clinical site (i.e., primary care centre) and rates of attendance at psychoeducational intervention. That is to say, we included both men and women from different primary care centres (from the total of eight) and with different degrees of adherence to the psychoeducational intervention (from 0 to 9 sessions). A study researcher contacted potential participants via telephone. Then, they were informed and invited to participate in the focus group. Participants involved in the focus groups were from distinct psychoeducational groups and they did not know each other. The script for the focus groups was based on the one used by Bair et al. (2009) and then refined according to the aims of the current study (see Table 2). Focus groups were held in meeting rooms of two different primary care centres with sessions lasting approximately 60 min. Both groups had the same moderator (CTP), with whom they had established a relationship prior to the study commencement in the psychoeducational sessions, and two observers (a female psychologist—ESR—and a female nurse) who took notes during the session paying special attention to non-verbal language and verbal coherence. No one else were present besides the participants and researchers. Sessions were audio-recorded and then professionally transcribed. Transcripts were not returned to participants for comment or correction neither provided feedback on the findings. Interviews were not repeated. At the beginning of the session all participants were informed that the session would be recorded for research purposes and were then asked to give their consent to participate in the session.

**TABLE 2:** Focus group script.

Pain and depression impact	1. What is the impact or difficulties pain causes in your life?
	2. What is the impact or difficulties depression causes in your life?
	3. What kind of difficulties do you have to cope or manage pain in a daily basis?
	4. What kind of difficulties do you have to cope or manage depression in a daily basis?
Utility of the psychoeducational program sessions	5. Which strategies from the psychoeducational program can you identify as useful to manage your pain? Why?
	6. Which strategies from the psychoeducational program can you identify as not useful to manage your pain? Why?
	7. Could you explain us what other strategies to manage or cope with your pain, depression or associated difficulties could be used (that have not been discussed previously)?
Barriers + Facilitators of the psychoeducational program	8. What obstacles can you identify in using these strategies?
	9. What factors do you believe can make these strategies easy to use regularly?
	10. Do you think mood problems, such as depression, made using these strategies more difficult?
Structure and content of the psychoeducational program	11. Do you believe the number of sessions was adequate?
	12. Do you believe the site of the sessions was adequate?
	13. Do you believe the duration of sessions was adequate?
	14. Do you believe the frequency of sessions was adequate?
	15. What would you change or think could be improved in the psychoeducational program so it could be more useful and effective? (topics/content, etc.)

### Individual semi-structured interviews

To improve the validity and richness of data collected from the focus groups, we decided to use semi-structured interviews. This data collection method is also an effective tool for gaining a deep understanding of participants’ experiences and perspectives regarding chronic health conditions (Pope et al., 2000). Semi-structured interviews are defined as an exploratory type-interview based on a flexible guide and focused on a main topic that it is to be explored (Magaldi and Berler, 2020). These interviews were found to be a suitable method to obtain data from individuals who were not interested in social interaction and had low adherence to the psychoeducational program. Moreover, conducting interviews via telephone provided greater flexibility. This contrasted with the focus groups, where participants were invited to gather face-to-face with other patients and engage in discussion and socialization. Initially, we had planned to conduct only focus groups to encourage discussion in a social context. However, in order to overcome the aforementioned issues, we had to consider adding another data collection method. Thus, this triangulation approach of informers (i.e., low vs. high adherent) and methodologies (i.e., focus groups and interviews) was used to validate the final themes and provide a higher level of assurance of unbiased interpretation. The interview was based on a framework of themes to be explored and prepared in advance (individuals interview’s questions were based on the focus group script; see Table 3). Inclusion criteria to participate in the telephonic interviews was low adherence participation to the psychoeducational intervention (i.e., only participants that attended 0–2 session). After five interviews we decided we reached relevant data (information was being repetitive) on the main barriers and facilitators to attend a psychoeducational programme and for that reason we decided it would be an appropriate number of participants. Interviews took between 20 and 30 min with an average of 23 min. As in the focus group, individual interviews were recorded with the participants’ permission after informed consent to participate.

**TABLE 3:** Individual semi-structured interview script.

1. Explain the phone call propose: to gather information on the factors that might influence the participation or non-participation in psychoeducational group sessions for chronic pain and depression.
2. What factors do you believe can represent an obstacle to participate in these type of group sessions?
3. How do you believe such obstacles can be overcome?
4. What factors do you believe can facilitate the participation in this type of sessions?
5. Would you like to leave any further comment or observation on this topic?

### Ethical considerations

All study procedures were approved by the Research Ethics Committee of the Jordi Gol i Gurina Primary Care Research Institute (IDIAP), Barcelona (Ref: P14/142). All participants gave their informed consent to participate in the original RCT and in this study.

### Data analysis

With the focus groups and individual interviews, we performed a content thematic analysis (Pope et al., 2000; Vaismoradi et al., 2016; Neuendorf, 2018). To analyse the data, several analytical steps were carried out: (1) four research team members (i.e., the four first authors—two females and two males) read each transcript independently and created a list of salient and significant participants’ quotes; (2) each researcher identified relevant themes by the creation of a preliminary list of themes reflected in the data; (3) identification of quotes and the preliminary themes were then reviewed and discussed among team members during weekly meetings to formalize an agreed upon code list. We used a mixed-strategy text codification based on previous research (Bair et al., 2009) and codes emerging from the data; (4) creation of preliminary categories grouping the codes by the criterion of analogy. Discrepancies in the choice of codes were resolved by consensus; (5) analysis of each final category, which was done independently by three members of the research team; and (6) drafting of a new text with the results. When analysing the transcripts, authors discussed data saturation and agreed that the data obtained in the two focus groups was enough to reach it. Data saturation was reached when researchers found out that all the needed data have been collected and there was not any new relevant information or data that could be collected from the participants (Guest et al., 2006; Fusch et al., 2018). To generate trustworthy and insightful findings, we solicited input from the research team members to capture similarities and differences in their perspective and generate unanticipated insights. By doing this, we aimed to guarantee the credibility, transferability, dependability and confirmability of the study’s findings (Nowell et al., 2017). Specific attention was given to themes marked by recurrence, repetition and emphasis (Owen, 1984). Data was summarized, grouped into conceptual themes (i.e., categories), and analysed based on standard qualitative research techniques (Crabtree and Miller, 1992). This study followed the Consolidated Criteria for Reporting Qualitative Research (COREQ) guidelines (Tong et al., 2007; see Supplementary material).

## Results

### Participants

Socio-demographic and clinical characteristics of the focus group participants (*N* = 10) are reported in Table 4. Participants’ age ranged between 51 and 72 years old (mean age = 63.5 years old), eight were females. Five participants were married or had a partner, two were single, two were widows and one was divorced. Six participants had primary education, two had secondary education, and two had high school education. Attendance to the psychoeducational sessions (nine in the total) ranged between 6 and 9 sessions with a mean of 7.7 sessions for this group.

**TABLE 4:** Sociodemographics and clinical features of participants.

Code	Sex	Age (years)	Depression severity	Pain intensity
**Focus group**				
M1	Male	58	1.75	7.25
W2	Female	67	2.05	6.75
W3	Female	65	0.95	5.50
W4	Female	68	1.25	5.75
W5	Female	51	1.55	10
M6	Male	55	2.25	6
W7	Female	69	2.30	4.75
W8	Female	72	1.45	5.25
W9	Female	71	2.25	5.75
W10	Female	59	1.80	9.50
**Individual interviews**				
W11	Female	77	2,25	8
W12	Female	61	2	5,50
M13	Male	43	1,02	7
W14	Female	71	1,25	6,7
M15	Male	67	1,35	5,30

Participants (*N* = 5) from the telephonic semi-structured interviews had a low adherence rate that ranged between 0 and 2 sessions with a mean of 0.8 sessions. Participants’ age ranged between 43 and 77 years old (mean age = 63.8 years old) and three were females. Four participants were married or had a partner and one was divorced. Three participants had primary education and two had secondary education, and only one was currently working (Table 4).

### Perceived barriers and facilitators to pain self-management

**FIGURE 1:**
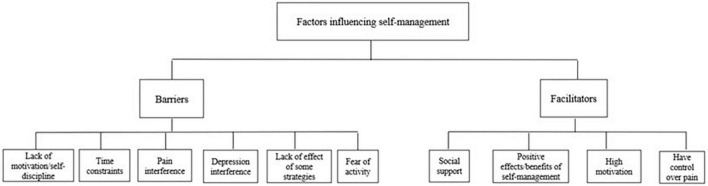
Coding tree for data analysis.

Focus groups conducted with the high adherent patients as well as interviews conducted with low adherent patients identified several barriers and facilitators to pain self-management (see Table 5 and Figure 1).

**TABLE 5:** Barriers and facilitators to use pain self-management strategies.

Barriers	Facilitators
Lack of motivation or self-discipline	Social support
Time constraints	Positive effects/benefits of self-management
Pain interference	High motivation/determination and self-discipline
Depression interference	Have control over pain
Lack of effect of some strategies	
Fear that activity worsens the pain problem	

#### Barriers to pain self-management

There were identified six categories regarding barriers for the use of pain self-management strategies (see Table 5) that are described below in order of frequency.

##### Lack of motivation or self-discipline

Most patients perceived lack of motivation/self-discipline as a key barrier to self-management adherence. Accordingly, poor intrinsic motivation to maintain self-management activities led to difficulties in adhering to them. They felt that their ability to do what needs to be done to improve their pain condition was largely compromised pulling them out from encouragement. Indeed, pain interference appears to compromise internal resources (e.g., motivation) to successful self-management: “*Before, I used to do yoga but now I do not do anything. I am very lazy*” (W2, high adherent). Nonetheless, they often felt they would improve their pain condition by engaging in such activities: “*When the doctor tells you that you have to do rehabilitation then you go, but when I am at home on my own, I keep postponing and postponing doing things that can help to improve my pain, and then the day goes by and nothing is done*” (M1, high adherent).

##### Time constraints

Patients believed that having no time was an important barrier to using self-management strategies. Indeed, self-management of chronic pain happens in a daily basis, and therefore the practical challenges such as the dedication of time to engage in certain activities can be perceived as a critical impediment: “*I do not have time to do it. I work 24 h a day*” (W10, high adherent); “*I have no time for these things (to gather in a group to discuss pain and do things)*” (W11, low adherent); “*I cannot find time for myself*…*I always have many things to do*” (M15, low adherent). Having to informally take care of someone (e.g., family member, close friend) was also felt as an obstacle to patients taking care of themselves. Accordingly, the sense of responsibility to take care of another person prevented them from being more active in self-managing their own pain condition: “*I do not have the time for these things since I have to take care of my grandchildren*” (W7, high adherent). Although patients commonly felt time constraints was a significant barrier, there was the perception that the problem was not time itself, but one’s motivation to engage in active self-management: “*The problem is not the time, but us, because it is always possible to find 5 min to do something*” (W4, high adherent).

##### Pain interference

The physical and psychological impact of pain was regarded as an important barrier to the use of self-management resources. Patients believed that pain negatively influenced their physical, social, and psychological functioning, and thus, feelings of powerlessness/frustration at the limitations caused by pain were commonly expressed by the patients. Indeed, chronic pain represented a barrier for increasing (physical or mental) or even to perform any activity level: “*If I did not have any pain in my knee I could go for a walk or engage in social activities, but since I have it how can I go out? What can I do? Should I cut my leg?*” (W5, high adherent); “*Pain limits my mental activities a lot*” (W7, high adherent); “*I have pain all the time* (…) *no energy to do things*” (W15, low adherent).

##### Depression interference

Patients felt that being in a negative mood and/or with significant depressive symptoms precluded them from engaging in active self-management. Self-management, which involves active participation of the patients themselves, was felt to be compromised by such depressive feelings. Those feelings were felt to impact their ability to proper problem-solving and adaptative coping with pain making it worse: “*When I am sad, I do not want to do anything. Now that I see myself like this, unable to do things, I get very sad and then I sink into the sofa. I lose my appetite, I start thinking about who I was before my pain problem, and all this makes my pain worse*” (W9, high adherent). Indeed, low motivation and initiative due to depression was perceived as a key barrier to participation in the psychoeducational programme as well as a barrier to adequate self-management: “*My depression does not let me do things. I have no joy.*” (W14, low adherent).

##### Other barriers to pain self-management

Although not widely mentioned by the high adherent patients, additional barriers to pain self-management were acknowledged, including the perceived lack of positive effects of self-management strategies. For example, some patients expressed fear that physical activity/exercise or any kind of activity would exacerbate their pain problem. Thus, due to fear of pain and/or reinjury, some patients were fear avoidant and perceived the use of certain strategies as not helpful. These beliefs prevented patients from engaging in active self-management.: “*When I exercise my pain gets worse*” (M1, high adherent); “*If it hurts already, it may hurt even more after exercising*” (W5, high adherent). Furthermore, low adherent participants pointed to the format of the psychoeducational programme, which was in group, and to no peer identification as obstacles to participate in self-management programs and strategies: “*I don’t want to talk about me in front of others, about my pain and stuff* (…). *I don’t like group sessions for that reason. I feel others judge me*” (W15, low adherent); “*In the first session I felt very awkward. I was the youngest one and all the others were very depressed. I felt this was not good for me, for my pain (.) to suffer in group*” (M13, low adherent).

#### Facilitators to pain self-management

Participants were able to identify factors that acted as facilitators in the use of self-management strategies (see Table 5). Four categories of facilitators were mentioned. The most common ones are presented below in decreasing order of frequency.

##### Social support

Support from family, friends or peers was the most common cited enabler that patients believed helped them to engage in pain relief strategies. Social support, i.e., support/social resources perceived by the patient to be available or provided within the context of informal (family, friends) or formal (peers, i.e., support group) relationships, was felt to play an important role in self-management. It appeared to increase patients’ self-efficacy, adaptative coping, and activation. Therefore, it encouraged them to become more proactive in implementing pain self-management practices. Family was viewed as a strong source of support: “*I have my husband and my son that understand my situation and are the only ones who give me support*” (W9, high adherent), as well as friends and peers: “*I need someone to cheer me up*” (W8, high adherent); “*The opinions of others also helped a lot*” (W9, high adherent).

##### Positive effects or benefits of self-management

Having benefits from using pain relief strategies was perceived as another important facilitating factor. According to the patients, such benefits emerged as a positive reinforcer to foster their pain self-management promoting high adherence to recommendations: “*If I have pain, I benefit from doing things that help my pain, so it is for my own benefit*” (W8); “*If it is something that works for me I do it and that’s it*” (W4).

##### High motivation and self-discipline

While lack of motivation and discipline was perceived as a barrier to self-management, the presence of high motivation and sense of self-discipline was perceived as a significant factor in improving self-management. Patient motivation was felt to be particularly relevant to active participation and promotion of adaptive pain self-management: “*Motivation and willpower to do it are important. If I cannot do it at 5 p.m., I will do it at 7 p.m., but I will do it. I have to find my time*” (W4, high adherent); “*You always have time to do it. You have to take your time*” (W7, high adherent).

##### Have control over pain

This facilitator was not widely discussed but emerged as relevant to some patients. The idea that chronic pain should not be avoided, but, instead, recognized and then controlled was central to some patients. Perceived control over pain refers to an individual’s belief about his/her own capacity of exerting influence on internal states, behaviors, and external environment. It represents a key protective factor for wellbeing as it fosters feelings of useful and worthwhile: “*I have to do things. I have to walk*” (W4, high adherent); “*If I stay at home, I feel useless, worthless. I must go out, see people, and feel I can do things*” (W2, high adherent).

##### Other facilitators to pain self-management

Additional facilitators to pain self-management were acknowledged by low adherent participants, such as clinical homogeneity of participants attending the intervention, and individual format of sessions: *“(*…*) In the group we should have similar mental health conditions, not just pain.*” (M13, low adherent); “*Individual sessions are better for me. I prefer to be alone*” (M15, low adherent).

### Psychoeducational intervention’s features (focus groups; *N* = 10)

We also aimed to gather information on the different aspects of our psychoeducational programme to understand its usefulness. As mentioned previously patients’ perceptions about the usefulness of the self-management program are scarce. Furthermore, some questions related to the following aspects were also included: usefulness, structure, content, and use of self-management strategies.

#### Usefulness

Regarding the usefulness of the psychoeducational programme, most patients felt that the program was an important source of social support. Patients mentioned that being in a support group led to positive effects permitting peer identification (i.e., having the same clinical condition), and free expression of feelings and thoughts. Participants believed peer support and positive effects were important ingredients to guarantee the usefulness of the sessions: “*What I found best in these sessions was the group, the people, and that you* [the professional] *dedicated time to explain things that were useful to us*” (W7). Several patients stated that being able to compare themselves with others helped them to put their pain in perspective. Being with similar others in a group intervention was felt to be empowering, as it gave a strong sense of normality and connection and helped patients to feel less isolated: “*The opinions of others also helped a lot*” (W9); “*These sessions were useful because we all have pain, and we did not feel alone*” (W2). Catharsis was cited as relevant since support group sessions gave the opportunity to freely and openly express emotions and thoughts. They expressed that being listened to in an empathic environment, peer validation and being motivated by others was truly valuable: “*The fact that we could talk helped us a lot*” (W8).

#### Structure

When asked about the sessions’ structure most patients commented that they would like to have a greater number of sessions. Patients mentioned that they would benefit from a higher number of sessions since it helped them managing pain and feeling good: “*We could have had more sessions as I felt really good and the things we learned helped me a lot*” (W9). Some mentioned that the duration of sessions should be shorter than 2 h due to their pain and associated discomfort. A patient shared that chairs should be designed around their needs and be more comfortable. Thus, ergonomics was revealed to be critical to the delivery of psychoeducational sessions, which has special importance in the delivery of assistance to pain patients: “*The chairs were very uncomfortable*” (W9).

#### Content

In relation to the sessions’ content participants mentioned it was appropriate. However, they suggested additional topics that were perceived as valuable for future self-management programmes. Patients felt the need to discuss the following topics as part of their worries regarding pain management: medication [“*It is important to understand the side effects of medication*” (M1)], the relationship between the health professional and the patient [“*We could had talked about the relationship we have with our doctor*” (W7)], sex and pain [“*It seems a taboo topic, but I think it would be important to talk about sex since it is really affected by pain*” (M6)], and suicide [”*Why didn’t we talk about suicide and despair? A person may think about killing herself/himself because she/he cannot stand the pain anymore*” (M6)].

#### Use of self-management strategies

During the psychoeducational sessions there were numerous pain control strategies that were discussed together with patients. Some of them were already part of the participants’ repertoire (as for example, physical activity or relaxation techniques), but others not. Most participants mentioned that the most useful learnt strategies for pain relief were those related to relaxation (e.g., breathing exercises), physical activity, attention management (i.e., distraction), and engagement in social activities. Positive benefits attained by practicing those strategies led to the reinforcement of adaptative self-management. The following statements illustrate those strategies’ use and benefits: “*I carry on doing the breathing exercises we learned in the sessions and it still helps me. I do it when I go to bed*” (M6); “*After the psychoeducational sessions I looked for a physical activity and I started my swimming classes, which helped with my pain. to improve it and forget it*” (W8).

## Discussion

Self-management is a complex and multifactorial process (de Ridder et al., 2008; Mann et al., 2017; Devan et al., 2018) influenced by several factors. This qualitative study aimed to gain a comprehensive understanding of how chronic pain patients with depression perceived, valued, and experienced pain self-management following a psychoeducational program that was previously tested (Aragonès et al., 2019).

This study found that factors that prevented patients from using self-management strategies were mainly related to the internal personal characteristics of the patients (e.g., lack of motivation) and pain-related issues (e.g., pain interference). Similar outcomes were found in previous research (Bayliss et al., 2003; Austrian et al., 2005; Jerant et al., 2005; Bair et al., 2009; Cuperus et al., 2013; Park et al., 2013; Lukewich et al., 2015; Matthias et al., 2016; Gordon et al., 2017; Mann et al., 2017; Devan et al., 2018) reporting both external and internal barriers. For example, in a study on veterans with musculoskeletal pain, it was found that the main barriers were related to motivation and engagement in the intervention (Matthias et al., 2016), while in other studies low-self-efficacy to manage pain (Mann et al., 2017) and pain interference (Bair et al., 2009) were revealed as the main barriers. In line with previous research, both the emotional and physiological effects of chronic disease were perceived as barriers to self-management (de Ridder et al., 2008). Moreover, low adherent participants, highlighted the format (group-based) of the psychoeducational sessions and no peer identification as the main obstacles preventing them to actively engage in self-management. Factors that facilitate self-management were related to the internal personal characteristics of the individuals (high motivation, having control over pain). Perceived facilitators reported by low adherent participants were related to personal characteristics, such as positive emotional state, less pain interference, but also related to features of the intervention itself, such as group patients having similar clinical characteristics, and time availability. Although less reported in the literature, facilitator factors found in this study support previous research on chronic pain self-management (Bair et al., 2009; Cuperus et al., 2013; Park et al., 2013; Matthias et al., 2016; Mann et al., 2017; Devan et al., 2018). For example, in adults with chronic pain positive attitude (Park et al., 2013), which includes, for example, high motivation, positive thinking and enjoyment of non-pharmacological pain management techniques (Wilcox et al., 2006), and self-confidence to manage pain (Mann et al., 2017) emerged as relevant facilitators.

Our study found that challenges and helpful factors for self-management in mixed chronic pain conditions, such as musculoskeletal pain, fibromyalgia or migraine, were more alike than different (Bair et al., 2009; Matthias et al., 2016; Devan et al., 2018; Takahashi et al., 2018). This finding suggests that self-management recommendations could potentially apply to different chronic pain conditions as they face similar challenges managing their pain. Furthermore, our study contributes to the existing literature by extending beyond previous reports that have largely focused on Anglo-Saxon countries.

The findings emphasize the complex and multifactorial nature of self-management. We believe that by including data from patients with varying levels of adherence, our study provides a more comprehensive understanding of the factors that influence self-management. Predictors of self-management were related to self-management delivery format (e.g., individual vs. group-based), for example, but also to psychological variables. Psychological variables, such as depression, motivation and *kinesiophobia*, were significant determinants of pain self-management and, thus, should be strongly addressed to ensure successful self-management. A recent study emphasized the role of such variables, including catastrophizing, in predicting multiple constructs of self-management (e.g., health-directed activities, skill and technique acquisition) (Banerjee et al., 2022).

Specifically, motivation was found to be the primary factor influencing self-management, which aligns with previous research (Choi et al., 2014; Jung and Jeong, 2016; Matthias et al., 2016). It plays a central role in determining how effectively patients learn to manage pain and use multiple pain coping strategies (Kerns et al., 2014; Jung and Jeong, 2016). Since self-management requires behavior change, motivation plays a vital role. Most theories and models of human behavior (e.g., social cognitive theories) consider behavior change to be primarily influenced by the perceived importance of behavior change and the belief that behavior change is possible (self-efficacy) (Bandura, 1986; Jensen et al., 2003). Such beliefs contribute to motivation, which in turn, affects behavior. That is well-posed by the motivational model for pain self-management, in which readiness to change behavior or motivation are essential to mediate the connection between a patient’s beliefs and her or his behavior (Jensen et al., 2003; Kratz et al., 2011). Despite motivation is a key predictor of effective self-management, there have been limited studies that explore the impact of motivational approaches intervention in self-management behavior (Molton et al., 2008; Kratz et al., 2011). The data underscore the importance of utilizing and incorporating into pain management treatments, a comprehensive model to understand motivation when pursuing effective self-management trials. *Kinesiophobia*, was another psychological variable revealed important in our study. In fact, it has also been pointed out as an important predictor of development and maintenance of chronic pain (Vlaeyen and Linton, 2000, 2012; Leeuw et al., 2007; Zale and Ditre, 2015; Gatchel et al., 2016), and self-management (Banerjee et al., 2022). It refers to fear-avoidance, which relates to the avoidance of movement or activities based on fear. Those dysfunctional interpretations can give rise to pain-related fear, and associated safety seeking behaviors such as avoidance/escape and hypervigilance, that can be a problem in long-lasting pain (Leeuw et al., 2007; Gatchel et al., 2016; Racine et al., 2017). In our sample, patients who fear that activity could worsen their pain condition avoided engagement in such activity. In addition, lower mood (i.e., depressive symptoms) was associated with poorer self-management. It appeared to hamper the patients’ ability to self-manage their pain or even to take initiative to adhere do self-management as found in previous literature (Bair et al., 2003; Dueñas et al., 2015; Zartaloudi et al., 2020).

Effective pain self-management involves patients taking an active role in managing their pain condition and being responsible for their own care (Stewart et al., 2014; Jonkman et al., 2016). Patients who participated in the psychoeducational intervention reported that self-management support was critical in learning coping skills to manage their chronic pain more effectively. This finding is not surprising considering previous research on self-management (Newman et al., 2004; Blyth et al., 2005; Devan et al., 2018), however, few pain self-management studies have conducted qualitative evaluations where patients explain, in their own words, what aspects of self-management they found valuable (Nees et al., 2020). Furthermore, the need to understand the patient’s preferences is essential to effective self-management as they are critical to promote a patient-centred care (PCC) perspective. PCC has been suggested as the number one recommendation for best musculoskeletal pain care (Lin et al., 2020) as it involves an emphatic and responsive approach to needs, values and expressed preferences of each individual patient (Rathert et al., 2012). Patients from this study reported that the psychoeducational intervention met their needs with clinical usefulness due to, for example, peer support and free expression of feelings/thoughts. We noticed that probably there was a dose-response in that attending a greater proportion of sessions led to better clinical outcomes. When looking at the different aspects of the intervention, such as the structure and content, patients reported that both were adequate, although they felt that some aspects could be improved for future interventions. Specifically, they pointed out the need to include additional topics pertinent in the context of chronic pain, such as medication side effects, sexual activity, health professional-patient relationship, and suicide. Studies reporting data on such needs are still scarce (Monga et al., 1998; Ambler et al., 2001; Cuperus et al., 2013; Racine et al., 2016) and thus warranted.

Data regarding the use and usefulness of pain self-management strategies from the patients’ views acknowledging such strategies is essential as they reflect an active process of decision-making that combines personal experiences with professional recommendations (Crowe et al., 2010). In this research, the most useful learnt strategies for pain relief were relaxation, physical activity, attention management (i.e., distraction), and engagement on social activities. Such pain self-management coping behaviors (Jensen et al., 2003) were associated with therapeutic benefits in our sample. Our findings are supported by a previous study, which found that preferred coping strategies were those that could be self-administered and included both physical and cognitive elements (Lansbury, 2000). Most common strategies used by participants to manage their chronic pain were medication, exercise, application of heat (Crowe et al., 2010) and resting (Blyth et al., 2005). However, they are contrary to the results reporting that the least-preferred strategies were the conventional treatments of medication, exercise and physiotherapy (Lansbury, 2000). Further studies are required to have a better understanding on the use and effect of specific self-management strategies on pain management.

The present study has some limitations that must be considered when interpreting the results. Participants of this study were mainly female patients. However, future research should attempt to focus on the perspective of male patients to increase the applicability of the study findings. Further, the results were based on the perspective of patients with musculoskeletal chronic pain and depression. Future studies focusing on healthcare professionals’ views in pain self-management are also required to complement the findings.

Despite the study’s limitations, an important strength of this qualitative study is that it is an in-depth analysis of a specific psychoeducational intervention tested previously (RCT) (Aragonès et al., 2019). It is a comprehensive reflection on the obstacles and facilitators that individuals with chronic pain and depression face when coping with pain. Enhancing patient self-management skills can improve pain-related outcomes (Von Korff and Moore, 2001; Damush et al., 2003; Dixon et al., 2007). Clinical implications relate to the need to address such information when dealing with chronic pain patients and when tailoring interventions, so that interventions become more effective by considering the factors that influence coping behavior. This may help healthcare professionals in their clinical encounters. Future research to determine whether patient centred interventions, motivation enhancement strategies or lower pain-related fear may increase active participation in pain self-management treatment programmes is warranted.

## Conclusion

The psychoeducational intervention was perceived as useful in promoting self-management. The findings show that pain self-management barriers and facilitators were mainly related to internal personal characteristics of the patients being similar among different cultural backgrounds and distinct chronic conditions. Thus, if confirmed, these data suggest that effective primary care self-management programmes for patients with both chronic musculoskeletal pain and depression would improve from focusing on these results. However, additional longitudinal and experimental studies are needed to evaluate the benefits of this possibility.

## Data availability statement

The raw data supporting the conclusions of this article will be made available by the authors, without undue reservation.

## Ethics statement

The studies involving human participants were reviewed and approved by the Research Ethics Committee of the Jordi Gol i Gurina Primary Care Research Institute (IDIAP), Barcelona (Ref: P14/142). The patients/participants provided their written informed consent to participate in this study.

## Author contributions

CT-P, EA, CR, GL-C, ES-R, AC, and JM contributed to conducting the underlying research (data collection and analyses) and drafting this manuscript. All authors contributed to the article and approved the submitted version.
